# Somatoform Disorders in Primary Care—An Exploratory Mixed-Methods Study on Experiences, Challenges and Coping Strategies of General Practitioners in the Federal Republic of Germany

**DOI:** 10.3390/ijerph21070901

**Published:** 2024-07-10

**Authors:** Julian Wangler, Michael Jansky

**Affiliations:** Centre for General Medicine and Geriatrics, University Medical Center of the Johannes Gutenberg University Mainz, Am Pulverturm 13, 55131 Mainz, Germany

**Keywords:** somatoform disorders, general practitioner, health anxiety, management

## Abstract

Somatoform symptoms are widely spread in outpatient care. For treating physicians, it can be challenging to establish a relationship that is conducive to compliance and to take stabilising action when dealing with affected patients. As primary care providers, GPs are usually the first point of contact for patients with somatoform disorders; they set the course for stabilisation and further care. To date, there is a lack of studies that focus on how GPs respond to such patients. In particular, strategies for establishing a stable doctor–patient relationship have hardly been explored. Consequently, this study investigated how GPs recognise the symptoms of somatoform disorders, what significance they attach to them and how they handle patients. The primary focus is on experienced patient properties, assumed causes of somatoform disorders, obstacles and complexities in consultation, care and stabilisation strategies, as well as diagnostic forms of support. A total of 2797 GPs in the German federal states of Hesse, Rhineland-Palatinate and Baden-Württemberg were surveyed anonymously by means of a written questionnaire between January and August 2023. A t-test was performed with independent samples to determine significant differences between two groups. In addition, 64 GPs were interviewed between March and April by means of qualitative semi-standardised interviews. The respondents make use of a wide range of communication and stabilisation strategies when treating somatoform physical complaints. The GPs combine the establishment of a tangential doctor–patient relationship with measures to consistently exclude physical causes and to enable the best possible assessment of patients, as well as to gently introduce them to the clinical picture of somatoform disorders. Most physicians are not familiar with current clinical guidelines. Cooperation with specialists and therapists is widely described as complicated. GPs have access to a wide range of communication and stabilisation strategies for the management of somatoform physical complaints. Yet, they experience interaction with this patient group as difficult in daily practice. GPs articulate a clear need for more external support. Apart from increasing therapeutic care capacities and interdisciplinary structures, it seems advisable to extend low-threshold therapy and support services.

## 1. Introduction

The prevalence of non-specific functional and somatoform physical complaints in primary care is stated as a proportion between 15 and 31 percent [1,2,3]. Such patients typically present to the general practitioner’s surgery with a wide variety of physical complaints without sufficient anatomical findings [4,5]. During the course of a long medical history, patients affected often have an exaggerated physical perception and show signs of psychological dysfunction [6,7,8]. Despite the lack of indications of a cause and repeated assurances by doctors that the signs of illness are not physically justifiable, patients affected are convinced of a physical cause. In comparison, the possibility of a psychological problem as the trigger of the complaints is usually dismissed [9]. As a matter of fact, apart from biological or genetic predispositions, there can be drastic and fundamental problems behind somatoform disorders, e.g., drastic life events, chronic diseases or serious illness experiences, social conflicts, lack of support, occupational stress, extreme body observation, health anxiety or hypochondria [10,11].

In the context of somatoform disorders, there are several problems involved in doctor–patient interaction [12,13]. Firstly, patients with unclear physical complaints often behave in an appellative manner that is determined by a persistent insistence on specific examinations (e.g., further diagnostics) [14]. Secondly, they meet with an understanding of their medical history from doctors, and a procedure that is intended to guarantee that no hidden illness is overlooked and that all potential trigger factors are included [15]. Thirdly, if anatomical causes cannot be found, there may be a discrepancy between the respective assumptions of the cause, which bears additional conflict potential [7]. If the doctor distinctly rules out anatomical causes and considers other potential explanations, the patient might feel misunderstood, accuse the doctor of professional incompetence and terminate the doctor–patient relationship [16,17,18].

An interdisciplinary S3 guideline on the clinical picture of somatoform disorders exists for German-speaking countries [3], whose recommendations for diagnostic and therapeutic issues are generally also suitable for the field of general practitioners. Furthermore, diagnostic instruments exist (e.g., International Diagnosis Checklist for ICD-10 ‘Somatoform Disorder’ or PHQ-15 for the assessment of the severity of somatic symptoms) [19]. With ICD-11 (implementation in Germany is expected to take place in 2027) and even more so DSM-5, the concept of somatoform disorders gradually changes. What is now important is not so much that the physical complaints have no explainable pathology, but rather that they are stressful for the patient.

While research projects on aspects such as prevalence [20] and helpful therapeutic strategies [21] have already been submitted for the German-speaking countries, there is a lack of studies that focus how general practitioners actually deal with these patients in daily practice [22]. Individual studies have been able to show that unspecific physical complaints without organic findings are widespread in general practitioners’ practices, and that this number has risen continuously in recent years. Unclear symptoms are often associated with pronounced health anxiety. In their everyday practice, which are short on time and resources, general practitioners are faced with the challenge of clarifying these often-complex symptoms and setting the course for appropriate further care [23,24]. In particular, communication strategies for establishing a stable doctor–patient relationship, which forms the basis for successful management, have hardly been researched to date.

### Research Interest

The aim of the study is to investigate how general practitioners recognise the symptoms of somatoform disorders in daily healthcare situations, what significance they attach to them and how they deal with affected patients. A special focus is placed on the determination of experienced patient characteristics, suspected causes of somatoform disorders, challenges experienced in consultation, care and stabilisation strategies, as well as diagnostic forms of support.

## 2. Material and Methods

### 2.1. Study Design and Survey Instrument

Quantitative survey: The explorative study was designed as an online survey, by means of which general practitioners were questioned. On the one hand, it is based on a comprehensive literature search. On the other hand, previous studies by the authors involving health anxiety were taken into consideration (e.g., [25,26]).

The designed survey instrument (see Supplementary Material, completion time: approx. 12 min) consists of the following components: (a) occurrence of somatoform disorders in daily practice; (b) patient characteristics; (c) diagnostic procedure and guideline orientation; (d) challenges experienced and stabilisation strategies; (e) interdisciplinary cooperation; (f) optimisation proposals.

In addition to the standardised questions, several open questions were included (6, 8, 16, 21). The socio-demographic features collected were gender, age, specialist background, practice environment, type of practice and number of patients per quarter. A pre-test was conducted with 15 randomly selected general practitioners prior to use in the field.

Qualitative interviews: The supplementary guideline for the semi-standardised interviews with general practitioners was deliberately based on the quantitative survey, although open questions were formulated here in accordance with the qualitative paradigm. In addition, the focus of the short interviews is on preferred strategies for the management of somatoform disorders.

### 2.2. Recruitment and Participants

Quantitative survey: Between January and August 2023, all 13,170 general practitioners actively treating patients in Hesse (3839), Rhineland-Palatinate (2667) and Baden-Württemberg (6664) received a written invitation to participate in the anonymised survey. The survey was a one-off letter in which the doctors to be surveyed were given password-protected access to the online survey. The participants did not receive any incentives.

Qualitative interviews: The recruitment of general practitioners for the qualitative interviews differed from that of the quantitative survey. First, a pool of 515 contact addresses was prepared with the help of the federal state-specific Internet doctor finder of the Association of Statutory Health Insurance Physicians, which contained a wide range of general practitioner practices in all federal states (30 to 35 addresses per federal state). For the German non-city states, it was ensured that all administrative districts were represented, that individual and joint practices occurred in approximately equal numbers, and that different practice environments were represented. Each federal state had to be equally represented in the study, regardless of the number of its inhabitants, in order to better represent rural regions in particular. When selecting the number of four general practitioners envisaged for each federal state, the following access criteria applied: at least 1 joint practice, balanced gender relationship, balanced relationship between urban and rural-small town practices, and consideration of older and younger doctors. A total of 82 general practitioner surgeries throughout Germany were contacted based on the deliberations described above. If interest in study participation was signalled, the doctors received study information. The recruitment period extended from December 2022 to March 2023. A total of 64 general practitioners were finally recruited for individual interviews.

All 64 interviews were conducted alternately by the authors between March and April 2023 in person orally, by telephone or via instant messaging service (30 to 45 min). The interviews were recorded.

### 2.3. Data Analysis

Quantitative survey: After cleansing the data set, the data were analysed using SPSS 23.0 for Windows. In order to determine significant differences between two groups, a t-test was used for independent samples (mean difference at the *p* < 0.001 level). This parametric method has a high-test strength and is considered to be statistically robust. The necessary conditions were met with the number of cases, the normal distribution of the groups to be distinguished and the fact that the samples come from the same population [27].

The evaluation of the open questions is based on post-coding in the sense of the qualitative content analysis [25].

Qualitative interviews: The first indications of theoretical saturation resulted after 48 interviews. The transcripts prepared after collection of the data were evaluated by the lead author with the help of a qualitative content analysis according to Mayring [25] (MAXQDA). The category system generated was closely oriented to the guideline and was repeatedly checked and, if necessary, modified as the evaluation continued. The pseudonymized codification of the interviews was carried out as follows: e.g., I-55-m (number of the interview; gender).

## 3. Results

### 3.1. Sample

Of the 2839 questionnaires processed, 2797 fully completed forms were included in the evaluation (response rate: 21%). Based on the socio-demographic characteristics collected, the sample is structured as follows:

- Gender: 53% male, 47% female;
- Average age: 54 (median: 53);
- Practice environment: 53% medium-sized and large cities, 47% rural-small towns;
- Type of practice: 52% individual practices, 44% joint practices, 4% other;
- Number of patients per quarter: 18% < 1000, 37% 1000–1500, 22% 1501–2000, 23% > 2000.

The sample obtained for the qualitative interviews is composed of:

- Gender: 22 male, 22 female;
- Average age: 55 (median: 55);
- Practice environment: 44 medium- and large-sized cities, 20 rural-small towns;
- Type of practice: 38 individual practices, 26 joint practices;
- Status: 48 practice owners, 16 employed doctors.

### 3.2. Somatoform Disorders in Everyday Practice

When asked about the assumed proportion of non-specific somatoform complaints in practice consultations, 43% stated a range between 16 and 20%, followed by 26% who assumed 10–15%.

Thirty-nine percent of all respondents assumed that the number of patients affected by non-specific somatoform physical complaints has been seen to increase in general practitioner care in recent years. Twenty-six percent stated that the proportion had increased moderately (twenty-five percent: not increased).

### 3.3. Patient Characteristics

With regard to various symptoms, 85% of respondents reported the occurrence of panic attacks and anxiety disorders among affected patients, followed by persistent pain such as headaches, chest or back pain (84%). In the experience of 72%, patients complained of fatigue and exhaustion, but also of cardiovascular (64%) and gastrointestinal (59%) complaints.

In patients with somatoform problems, the respondents observed an increased frequency of consultation in connection with numerous questions about their own complaints, as well as the demand for instrument-based diagnostics (cf. Table 1). The respondents also frequently stated false or exaggerated expectations regarding the services to be provided by the general practitioner. Doctors often noticed an extreme sensitivity and nervousness in patients. Once patients were convinced that they know the cause of their illness, the respondents experienced difficulty in distracting them from their opinions and concerns.

When it comes to suspected or actual causes for the development of somatoform disorders, the respondents primarily stated extraordinarily stressful situations, such as unemployment, separation, accidents, operations or the loss of a close person (85%), but also stress and conflicts at work (80%). A routinely anxious way of handling physical complaints or hypochondria (71%) was also mentioned.

### 3.4. Diagnostic Procedure and Guideline Orientation

At 30%, a smaller proportion coded in the area of neurotic stress and somatoform disorders as per ICD. Of these, 21% in turn stated that they do so frequently, and another 28% occasionally. Another part of the respondents stated that they confine themselves to the description of comorbidities such as depression, anxiety disorders and/or diagnoses such as unclassified chronic pain.

In an enquiry, reasons were identified as to why the ICD code is hardly used for this clinical picture. At the centre of this is the fact that the general practitioners questioned considered an early determination of the ICD code to be premature and little differentiated in view of such a complex ailment.

*“The ICD terminology is too specific and requires a quick determination. Can I be sure that it is not in fact depression or some other problem?”*

*“It is often difficult to be sure what is actually behind such complaints and how they might interact with each other. So, I avoid ICD.”*

In this regard, there is too little knowledge of the content of somatoform physical complaints, so that a determination of the ICD code seems rushed. Many respondents did not claim to make a specific diagnosis but concentrated on excluding common physical causes to a large extent.

*“For me, my work ends where I find no indication of a clear physical cause.”*

Other respondents state the lacking consistency of coding.

*“Nothing is achieved by categorising the patient.”*

*“This is all about holistic medicine,* i.e., *looking at the patients in all their complexity and helping them as well as possible. An ICD code is only a label.*”

A follow-up question revealed that only 15% of the doctors questioned the use of certain diagnostic tools such as (psychosomatic) checklists or forms for diagnostics or for differential diagnostic deliberations for this clinical picture. An analogous picture emerges with regard to the interdisciplinary S3 guideline on somatoform disorders. Twenty-eight percent of the respondents stated that they are aware of this.

Thirty-nine percent of the doctors familiar with the S3 guideline assessed it as being quite useful. In an open question, numerous respondents criticised the guideline: “*inconvenient and impractical*”, “*too distant from application*”, “*no individual consideration of the patient possible*”, “*does not do justice to the complexity of psychosomatic disorders*”.

### 3.5. Challenges and Stabilisation Strategies

Sixty-five percent of all respondents experienced the management of patients with non-specific somatoform physical complaints as very or rather strenuous in their daily practice (twenty-seven percent less or not at all strenuous). The proportion of doctors who generally experience challenges was clearly higher among doctors in small towns and rural communities than in medium- and large-sized cities (76% to 54%, *p* < 0.001).

With regard to dealing with somatoform patients, the doctors questioned experienced the most varied challenges (cf. Table 2). In addition to the problem of providing sufficient time for consultation, a clear majority of the respondents find it rather difficult to influence these patients in such a way that excessive fear of the presence of a serious illness can be prevented and misunderstandings avoided. Closely linked to this is ensuring compliance. About three quarters of those questioned find it challenging to motivate patients to make use of appropriate psychosocial support.

Despite the requirements and problems involved with somatoform complaints, the respondents state numerous strategies with which they have had positive experiences in stabilising patients (cf. Table 3). Most of the respondents articulated that they consider in-depth and continuous intercommunication with the patient in a relaxed conversation situation to be particularly important to enable the development of a trusting doctor–patient interaction at eye level. A large part of the doctors attached importance to assessing the patient’s background and life situation.

The restraint exercised during tangential discussion is intended to avoid raising false expectations, provide rational clarification and avoid excessive examinations. This may possibly require an early relativisation of inappropriate wishes and requests. Regarding their approach, the respondents attempt to address psychosocial topics rather casually at first. In the opinion of the respondents, regular appointments, i.e., appointments that are independent of complaints and fears, should be arranged for a limited period of time. In order to introduce patients to the clinical picture of somatoform disorders, information material is sometimes compiled. Complaints diaries are provided to help determine more precisely under which everyday conditions the symptoms occur.

Apart from the reference to relaxation techniques, several doctors emphasised the importance of regular physical activity as a therapeutic instrument, e.g., to change awareness routines and support the retrieval of subjectively perceived sovereignty in daily life. Most of the respondents were clearly hesitant to treat somatoform disorders with medication.

### 3.6. Qualitative Findings

In the interviews, most of the general practitioners interviewed also made it clear that they attach particular importance to an in-depth and continuous interaction with the patient in a dialogue situation that is as relaxed as possible. In their opinion, a relaxed, supportive and empathetic attitude, as well as a patient-centred discussion, are of paramount importance for a trusting doctor–patient interaction. When obtaining information, the doctor should ensure to treat the somatoform patient as a partner at eye level, i.e., listen to his/her complaints in detail and address them without reservations. For some doctors, it is important to ask questions at an early stage to help them assess the patient’s background, life situation and personality (e.g., high level of body awareness, hypersensitivity), as this may be relevant for further management and the assessment of therapeutic options.

*“Not everyone needs a therapist right away, and that is not really desirable anyway. Sometimes, completely different approaches help reach the goal in the long run.”*

*(I-11-m)*

The restraint exercised during tangential discussion is intended to avoid raising false expectations, provide rational clarification and avoid excessive examinations. This may possibly require an early relativisation of inappropriate wishes and requests, but in a way that the patient himself/herself is more likely to realise which expectations are realistic and which are not. With regard to their approach, the doctors interviewed reported that they attempt to address psychosocial issues in a casual and indirect way, using terms from daily life. They considered this a critical point, because if patients have the feeling of being “pushed into a psychological category” at an early stage, they may feel that their concerns are not being taken seriously. The following is, therefore, important:

*“It is important to help the patient to get to know himself/herself better and to have an open view of all the stress factors. That is what I would also consider as basic psychosomatic care, where we general practitioners are in a position to help well”.*

*(I-23-f)*

In the opinion of the respondents, regular appointments, i.e., appointments that are independent of complaints and fears, should be arranged for a limited period of time. Apart from realistic and possibly verifiable discussion and therapy targets, some of the doctors emphasised that one should “not do too much in too short a time”. This leads to a tense situation for both doctor and patient.

*“I think it is particularly important at an early stage to keep signalling to the patient that he/she is in the right place here. This includes acknowledging small steps and successes, especially because these patients often have very sensitive personalities.”*

*(I-39-f)*

Regarding specific measures for the management of somatoform patients, some of the respondents first of all emphasise the importance of continuously working on the “awareness of the patients […] that there are symptoms that occur without a clear physical cause”. In some cases, information material is compiled for this purpose. Complaints diaries are intended to help isolate symptoms and determine more precisely under which everyday conditions these symptoms occur.

*“On the one hand, this signals that you take your dialogue partner seriously, and on the other hand, it actually helps to seek the causes, such as pain, a little more precisely”.*

*(I-55-m)*

Apart from referring to relaxation techniques, several doctors emphasised the importance of regular physical activity as a therapeutic instrument. This is not just about moving awareness routines, but about supporting the retrieval of subjectively perceived sovereignty and a sense of control in daily life.

*“Anxious body observation and hypersensitisation lead to increased tension and thus to increased discomfort. Increasing self-efficacy is therefore central.”*

*(I-31-m)*

In the course of the development of compact online therapy offered by certain health insurance companies, some general practitioners are trying to introduce somatoform patients to such offers where psychosocial care appears to be necessary. The idea is that patients often resist standard therapies but may possibly accept a low-threshold, anonymous offer of help.

*“I have had good experiences with this. Online therapies are a real alternative to help certain patient groups.”*

*(I-7-m)*

Most of the interviewed general practitioners usually avoided a medication-based treatment of somatoform disorders. Only in individual cases, in which psychopharmacological interventions exist, for example due to simultaneous depression, anxiety, obsessive–compulsive or sleep disorders, are attempts made to include medication-based solutions. Here, it is preferably attempted to treat occurring pain, sleep disorders and depressive symptoms simultaneously, if possible, with a single medication. In this context, however, the respondents insist on a functioning and closely scheduled interdisciplinary cooperation.

### 3.7. Interdisciplinary Cooperation

Regarding interdisciplinary cooperation, only some of the respondents had the impression that they can rely on cooperation with regional physicians for diagnostic clarification and/or treatment of somatoform complaints. Forty percent felt that the cooperation in this area is very good or rather good, whereas fifty percent considered it as rather poor or very poor. While 55% of the physicians in large- and medium-sized cities assessed the cooperation with colleagues from psychiatry/psychosomatics and neurology as good, only 26% of physicians in small towns and rural communities were of the same opinion (*p* < 0.001). A similar picture emerges with regard to cooperation with psychotherapists. Forty-two percent considered this as very good or rather good, while fifty-one percent rated it as rather poor or very poor.

A follow-up question provided information on the causes of the dissatisfaction established. Thus, 41% considered that the specialist colleagues in psychiatry and psychotherapy usually provide sufficient information about the examinations, results and/or therapeutic measures performed. In the case of psychotherapists, 65% stated that due to long waiting times, they have become rather reluctant as regards referrals and rather seek other solutions, if necessary. Seventy-five percent stated that they often feel left alone in the management of patients with somatoform complaints. While the proportion among urban doctors was 61%, it was 88% among rural doctors (*p* < 0.001).

### 3.8. Optimisation Suggestions

With a view to the potential optimisation of the care of somatoform physical complaints, some of the respondents (47%) named the creation of significantly more psychotherapeutic therapy places. This was followed by various suggestions intended to anchor health insurance companies more strongly as contact persons for psychosomatic problems (35%).

The availability of online therapy services, whether provided by certain health insurance companies or in the form of digital health applications, was considered to be promising. Some of the respondents (32%) attempt to introduce somatoform patients to such services where psychosocial care appears to be necessary. The idea is that patients often resist standard therapies but may possibly accept a low-threshold, anonymous offer of help.

## 4. Discussion

### 4.1. Main Findings and Comparison with Previous Studies

The findings of the quantitative and qualitative survey show that in the perception of general practitioners, somatoform disorders make up a considerable and increasing proportion of patient consultation. Patients with somatoform disorders have a characteristic behavioural profile that may pose challenges for general practitioners [4,6,7]. Therefore, patients affected can trigger reservations, negative positioning and conflictual attitudes in general practitioners [23,28]. In light of the findings, it is evident that general practitioners bear potential psychological causes and intensifying stress factors in mind.

The quantitative and qualitative findings are closely related and can be considered jointly in almost every respect. The results show that general practitioners consider a stable doctor–patient relationship to be a central prerequisite for successful treatment. Therefore, they make various efforts to establish a resilient, tangential basis for consultation [29,30]. This is accompanied by measures to consistently exclude physical causes on the one hand and to enable the best possible assessment of patients, as well as to gently introduce them to the clinical picture of somatoform disorders, on the other. The general practitioners questioned mainly attach importance to providing sufficient consultation time for this group of patients. It is worth noting that in English-language studies, such as Wortman et al., similar points are raised, including ‘Continuously searching for common ground’ and ‘Making patients aware of the interaction between body and mind’ [31]. As Stone already observed, general practitioners may experience the interaction with patients affected by physically unexplained complaints as personally and ethically enhancing, as the image of holistic, speaking medicine can fully develop here [32]. In addition, the approaches selected show parallels to the treatment of patients with health anxiety, who are associated with the phenomenon of cyberchondria [23,25,26].

Knowledge and application of the S3 Guidelines was surprisingly low; there were reservations and criticism among the respondents as regards their applicability. A major problem seems to be a lack of practical relevance and over-complexity [16,32,33]. In the paper by Natschke et al., the attitude of general practitioners with regard to the S3 guideline on somatoform complaints was also critical [29]. The authors then proposed the preparation of a user-friendly version for general practitioners.

However, it seems questionable whether such a practice-oriented, compact version will change the basic finding that general practitioners tend to avoid diagnostic guidelines and tools for somatoform disorders and proceed according to their personal understanding of the case in question [16]. An explicit diagnosis as a somatoform disorder is obviously not experienced as helpful in dealing with the symptoms, similar to depression [34,35,36]. This indicates that general practitioners experience a diagnosis as a potential problem in dealing with patients, and that the background of the occurrence cannot be clarified due to the individual differences of somatoform problems [30]. Earlier studies were also able to prove that questionnaires, for example, are only used to a limited extent as screening instruments in GP surgeries [1,2,8,25,37,38,39]. Coding according to ICD, therefore, can be described as challenging, as numerous overlapping diagnoses are described [19,40,41].

General practitioners experience various difficulties at the interfaces of interdisciplinary cooperation with colleagues from psychiatry/psychosomatics and neurology, as well as with psychotherapists. Long waiting times and communication problems (e.g., lack of direct case-related exchange) mean that general practitioners can often only rely on themselves. Consistently noteworthy in the findings of this study is that rural doctors experience the care of somatoform disorders as a greater challenge. This may be related to the fact that doctors outside urban centres can rely far less on an adequate care and support network. This shows the necessity for better cooperation and the strengthening of interdisciplinary structures [8,42,43]. In the opinion of the doctors questioned, low-threshold solutions, such as online therapies, can help to compensate for the lack of psychosomatic care capacities in somatoform disorders. Here, the statutory health insurance companies in particular can play a more proactive role [44]. The development of new offers, such as digital health apps, represents a major opportunity in this context.

### 4.2. Strengths and Limitations

Although it was possible to obtain a large, heterogeneous sample of primary care providers, various limitations of the survey must be reflected. These include a regional recruitment focus in three federal states and a limited response (quantitative survey). Moreover, it is possible that general practitioners with an interest in the subject may have shown stronger participation in the survey (selection bias).

This study was only a cross-sectional study and only correlative relationships can be uncovered but—due to the lack of longitudinal data—not robust causal relationships.

## 5. Conclusions

The results show that general practitioners consider a good and stable doctor–patient relationship as a central basis for successful treatment. General practitioners apply a wide range of communication and stabilisation strategies in the care of somatoform physical complaints. Therefore, they demonstrate competence and important prerequisites for mastering the communicative challenges that are to be expected in the treatment of somatoform disorders. Nevertheless, contact with this group of patients is experienced as a particular challenge in daily practice. In the long run, apart from the creation of more therapeutic care capacities and interdisciplinary structures, it would be helpful to strengthen low-threshold therapy and support services.

## Figures and Tables

**Table 1:** Characteristics of patients with somatoform complaints.

Question: In your opinion or experience, which of the following characteristics apply frequently to patients affected by somatoform physical complaints/disorders? (N = 2797; multiple entries)	
Patients ask many questions	96%
Patients seek medical consultation more often than the average	95%
Patients often request further instrument-based diagnostics	86%
Patients come for consultation with false expectations or assumptions (e.g., information consulted, general practitioner role)	86%
Patients are anxious, nervous, easily become worked up	84%
Overworked, occupational stress	81%
Excessively sensitive, easily irritated, quickly feel offended	79%
Patients are easily influenced by other sources of information	77%
It is difficult to distract patients from their opinions, worries or concerns.	76%
Patients are frequently affected by psychological disorders such as depression, panic or obsessive–compulsive disorders	75%
Patients are more critical towards me as a doctor	55%
Patients quickly break off contact with the doctor if their expectations are disappointed.	53%
Patients suffer from a chronic disease	48%
Patients are aggressive, ready for conflict	44%
Patients tend to imagine physical complaints	42%
Patients have little trust, are sceptical	41%
These patients lack realisation of their actual condition	39%
Patients tend to practice self-medication	38%
Patients have an exaggerated use of medication	37%

**Table 2:** Experienced challenges in dealing with patients with somatoform complaints.

Question: Doctors may be faced with challenges in the care and treatment of patients with non-specific somatoform physical complaints/disorders. How great do you experience the following potential challenges when you think about your previous experiences with these patients? (N = 2797; categories ‘Very challenging/Rather challenging’ and ‘Less challenging/Not at all challenging’ combined)	
Providing sufficient time for these patients	90%/10%
Ensuring compliance	89%/11%
Avoiding or eliminating misunderstandings and disappointments on the part of the patients	85%/15%
Counteracting or eliminating concerns or fears of a possible illness	85%/15%
Responding to all questions and wishes of these patients (e.g., with regard to instrument-based diagnostics)	83%/17%
Providing patients with a realistic picture of the possibilities and limitations of medical diagnostics and/or therapy	80%/20%
Encouraging patients to make use of psychosocial support services (e.g., psychotherapy, resilience training)	75%/25%

**Table 3:** Strategies in the management of somatoform disorders with the aim of stabilising the patient and the doctor–patient relationship.

Question: Here are various imaginable approaches that the general practitioner can apply to stabilise patients with non-specific somatoform physical complaints/disorders or to have a positive influence on them, so that the doctor–patient relationship also benefits from this. Which of these have you already applied and experienced a good result with? (N = 2797; multiple entries)		
Dimension: Dialogue	Tangential dialogue: Doctor–patient dialogue follows the report of the patient’s complaints; the patient is given space to explain; confrontational dialogue techniques are avoided; radiation of a calm, objective attitude	92%
	Avoidance of inciting exaggerated expectations regarding diagnostics and therapy; dampening of over-optimistic patient expectations	87%
	Assurance of the credibility of the complaints	83%
	Getting to know the patient to enable the assessment of his/her personality	73%
	Careful marking of references to psychosocial problems as relevant and addressing them in a casual manner (“stress”, “strain”, etc.)	71%
	Refraining from negative wording of diagnostic findings (“You aren’t ill”)	70%
	Long-term building of motivation for holistic treatment if possible	70%
Dimension: Treatment framework and goals	Provision of more consultation time (e.g., detailed explanation, emotional support)	91%
	Scheduling of regular appointments (time-contingent instead of complaint-driven)	80%
	Targeting in intermediate steps; not too many goals in too short a time	78%
	Formulation of realistic, specific and verifiable therapy goals (improvement of quality of life instead of targeting complete cure)	75%
Dimension: Therapeutic measures and strategies	Use of selected information material to successively communicate to the patient that there are symptoms without a clear physical origin	75%
	Physiotherapy services	66%
	Prescription of supporting measures, e.g., procedures for relaxation and stress management, mindfulness training	62%
	Diary of complaints or anxieties: When do complaints occur and in what way?	59%
	Dosed physical activity to change body awareness (reduction of fear and loss of control)	58%
	Referral of the patient to a psychotherapist	48%
	Referral to low-threshold psychosocial or psychotherapeutic services (e.g., special services offered by health insurance companies)	45%
	Referral or arrangement of psychosocial support services (e.g., consultation centres, self-help groups)	42%
	Medication therapy preferably only for pronounced comorbidities	29%

## Data Availability

All major data generated or analysed during this study are included in this published article. Additional information can be provided on request made to the corresponding author.

## Supplementary Materials

The following supporting information can be downloaded at: https://www.mdpi.com/article/10.3390/ijerph21070901/s1, File S1: Survey questionnaire: Non-specific somatoform physical complaints/disorders in GP practices.

## Abbreviation

GP(s): General Practitioner(s).

## References

[1] Fink P., Hansen M.S., Oxhøj M.L. (2004). The prevalence of somatoform disorders among internal medical inpatients. J. Psychosom. Res..

[2] Wittchen H.U., Jacobi F., Rehm J., Gustavsson A., Svensson M., Jönsson B., Olesen J., Allgulander C., Alonso J., Faravelli C. (2011). The size and burden of mental disorders and other disorders of the brain in Europe 2010. Eur. Neuropsychopharmacol..

[3] Schaefert R., Hausteiner-Wiehle C., Häuser W., Ronel J., Herrmann M., Henningsen P. (2012). Clinical Practice Guideline: Non-specific, functional and somatoform bodily complaints. Dtsch. Arztebl. Int..

[4] Sharma M.P., Manjula M. (2013). Behavioural and psychological management of somatic symptom disorders: An overview. Int. Rev. Psychiatry.

[5] Oyama O., Paltoo C., Greengold J. (2007). Somatoform disorders. Am. Fam. Physician.

[6] Chaturvedi S.K., Desai G., Shaligram D. (2006). Somatoform disorders, somatization and abnormal illness behaviour. Int. Rev. Psychiatry.

[7] Khan A.A., Khan A., Harezlak J., Tu W., Kroenke K. (2003). Somatic symptoms in primary care: Etiology and outcome. Psychosomatics.

[8] Le T.L., Mylopoulos M., Bearss E., Geist R., Maunder R. (2022). Multiple symptoms and health anxiety in primary care: A qualitative study of tensions and collaboration between patients and family physicians. BMJ Open.

[9] Guo D., Kleinstäuber M., Johnson M.H., Sundram F. (2019). Evaluating Commonalities Across Medically Unexplained Symptoms. Int. J. Environ. Res. Public Health.

[10] Grover S., Aneja J., Sharma A., Malhotra R., Varma S., Basu D., Avasthi A. (2015). Do the various categories of somatoform disorders differ from each other in symptom profile and psychological correlates. Int. J. Soc. Psychiatry.

[11] Dohrenwend A., Skillings J.L. (2009). Diagnosis-specific management of somatoform disorders: Moving beyond “vague complaints of pain”. J. Pain.

[12] Jablensky A., Ono Y., Janca A., Asai M., Sartorius N. (1999). The concept of somatoform disorders: A comment on the mind-body problem in psychiatry. Somatoform Disorders. A Worldwide Perspective.

[13] Stuart S., Noyes R. (1999). Attachment and interpersonal communication in somatization. Psychosomatitics.

[14] Weiss F.D., Rief W., Kleinstäuber M. (2017). Health care utilization in outpatients with somatoform disorders: Descriptives, interdiagnostic differences, and potential mediating factors. Gen. Hosp. Psychiatry.

[15] Leutgeb R., Berger S., Szecsenyi J., Laux G. (2018). Patients with somatoform disorders: More frequent attendance and higher utilization in primary Out-of-Hours care?. PLoS ONE.

[16] Hartz A.J., Noyes R., Bentler S.E., Damiano P.C., Willard J.C., Momany E.T. (2000). Unexplained symptoms in primary care: Perspectives of doctors and patients. Gen. Hosp. Psychiatry.

[17] Hüsing P., Löwe B., Piontek K. (2018). Somatoform disorder in primary care: The influence of co-morbidity with anxiety and depression on health care utilization. J. Eval. Clin. Pract..

[18] Sharpe M., Mayou R. (2004). Somatoform disorders: A help or hindrance to good patient care?. Br. J. Psychiatry.

[19] Wangler J., Jansky M. (2022). What can diagnostic algorithms do for primary care?—Results of a survey among general practitioners in Germany. Prim. Hosp. Care.

[20] Haller H., Cramer H., Lauche R., Dobos G. (2015). Somatoform disorders and medically unexplained symptoms in primary care—A systematic review and meta-analysis of prevalence. Dtsch. Arztebl. Int..

[21] Henningsen P., Zipfel S., Herzog W. (2007). Management of functional somatic syndromes. Lancet.

[22] Margo K.L., Margo G.M. (1994). The problem of somatization in family practice. Am. Fam. Physician.

[23] van der Weijden T., van Velsen M., Dinant G.-J., van Hasselt C.M., Grol R. (2003). Unexplained complaints in general practice: Prevalence, patients’ expectations, and professionals’ test-ordering behavior. Med. Decis. Mak..

[24] van Bokhoven M.A., Koch H., van der Weijden T., Grol R.P.T.M., Kester A.D., Rinkens P.E.L.M., Bindels P.J.E., Dinant G.-J. (2009). Influence of watchful waiting on satisfaction and anxiety among patients seeking care for unexplained complaints. Ann. Fam. Med..

[25] Wangler J., Jansky M. (2020). General practitioners’ challenges and strategies in dealing with Internet-related health anxieties—Results of a qualitative study among primary care physicians in Germany. Wien. Med. Wochenschr..

[26] Wangler J., Jansky M. (2023). Enquiries and health concerns—A survey of German general practitioners regarding experiences and strategies in patient care. J. Public Health.

[27] Bortz J., Schuster C. (2010). Statistik für Human-und Sozialwissenschaftler [Statistics for Human and Social Scientists].

[28] Natschke T., Heintze C., Döpfmer S. (2016). Patients with Non-specific Somatoform Disorders in Primary Care Practice—A Qualitative Survey among Family Practitioners. Z. Allg. Med..

[29] Rief W., Sharpe M. (2004). Somatoform disorders—New approaches to classification, conzeptualization, and treatment. J. Psychosom. Res..

[30] Janca A. (2005). Rethinking somatoform disorders. Curr. Opin. Psychiatry.

[31] Wortman M.S.H., Hartman T.C.O., van der Wouden J.C. (2022). Perceived working mechanisms of psychosomatic therapy in patients with persistent somatic symptoms in primary care: A qualitative study. BMJ Open.

[32] Stone L. (2013). Reframing chaos—A qualitative study of GPs managing patients with medically unexplained symptoms. Aust. Fam. Physician.

[33] Bishop F.L., Dima A.L., Ngui J., Little P., Moss-Morris R., Foster N.E., Lewith G.T. (2015). “Lovely pie in the sky plans”: A qualitative study of clinicians’ perspectives on guidelines for managing low back pain in primary care in England. Spine.

[34] Harkness E.F., Harrington V., Hinder S., O’brien S.J., Thompson D.G., Beech P., Chew-Graham C.A. (2013). GP perspectives of irritable bowel syndrome—An accepted illness, but management deviates from guidelines: A qualitative study. BMC Fam. Pract..

[35] Jobst D. (2006). Familiy Doctors—How Would They Diagnose a Somatoforme Disorder?. Z. Allg. Med..

[36] Kammerer K., Falk K., Döpfmer S., Heintze C. (2018). GPs’ Perceptions of Strengths and Shortcomings of the ICD-10 for Diagnosis of depression. Gesundheitswesen.

[37] Fink P., Rosendal M., Olesen F. (2005). Classification of somatization and functional somatic symptoms in primary care. Aust. N. Z. J. Psychiatry.

[38] Janca A., Isaac M., Ventouras J. (2006). Towards better understanding and management of somatoform disorders. Int. Rev. Psychiatry.

[39] Looper K.J., Kirmayer L.J. (2002). Behavioral medicine approaches to somatoform disorders. J. Consult. Clin. Psychol..

[40] Budtz-Lilly A., Schröder A., Rask M.T., Fink P., Vestergaard M., Rosendal M. (2015). Bodily distress syndrome: A new diagnosis for functional disorders in primary care?. BMC Fam. Pract..

[41] Fink P., Sørensen L., Engberg M., Holm M., Munk-Jørgensen P. (1999). Somatization in primary care. Prevalence, health care utilization, and general practitioner recognition. Psychosomatics.

[42] Jorsh M.S. (2006). Somatoform disorders: The role of consultation liaison psychiatry. Int. Rev. Psychiatry.

[43] Strassnig M., Stowell K.R., First M.B., Pincus H.A. (2006). General medical and psychiatric perspectives on somatoform disorders: Separated by an uncommon language. Curr. Opin. Psychiatry.

[44] Eichenberg C., Schott M. (2019). Use of online health services in individuals with and without symptoms of hypochondria: Survey Study. J. Med. Internet Res..

