# Supplementary material for: Somatoform Disorders in Primary Care—An Exploratory Mixed-Methods Study on Experiences, Challenges and Coping Strategies of General Practitioners in the Federal Republic of Germany
Source: Int J Environ Res Public Health. 2024 Jul 10;21(7):901. doi: 10.3390/ijerph21070901 (PMC11277205; doi:10.3390/ijerph21070901)
Supplement: Supplementary file 1 [file ijerph-21-00901-s001.zip › Supplementary material.pdf]

1. Would you say that the number patients affected by non-specific somatoform physical complaints/disorders has been seen to increase in GP care in recent years, or would you not?

◯ Yes, strongly increasing ◯ Yes, somewhat increasing ◯ No, not increasing
◯ Hard to say, don't know

2. What do you estimate: What is the total proportion of medical consultations in your practice that are caused by non-specific somatoform symptoms or in which somatoform symptoms play a significant role?

◯ Less than 10% ◯ 10-15% ◯ 16-20% ◯ 21-25% ◯ 26-30% ◯ More than 30%
◯ Hard to say, don't know

3. How often do the following symptoms occur in your patients who are affected by somatoform physical complaints/disorders?

Persistent pain (e.g. headache, chest or back pain)
◯ Frequently ◯ Occasionally ◯ Rarely ◯ Never

Fatigue, exhaustion
◯ Frequently ◯ Occasionally ◯ Rarely ◯ Never

Dizziness
◯ Frequently ◯ Occasionally ◯ Rarely ◯ Never

Breathing disorders (e.g. feeling of inhibition of breathing, tightness of the throat, shortness of breath)
◯ Frequently ◯ Occasionally ◯ Rarely ◯ Never

Difficulty swallowing, lump in the throat
◯ Frequently ◯ Occasionally ◯ Rarely ◯ Never

Gastrointestinal complaints (e.g. sensitive stomach, irritable bowel, nausea, feeling of fullness, abdominal pain)
◯ Frequently ◯ Occasionally ◯ Rarely ◯ Never

Cardiovascular disorders (e.g. feeling of pressure, stitches, feeling of tightness in the chest, palpitations)
◯ Frequently ◯ Occasionally ◯ Rarely ◯ Never

Panic attacks, anxiety disorders
◯ Frequently ◯ Occasionally ◯ Rarely ◯ Never

Other, namely: ________________________
◯ Frequently ◯ Occasionally ◯ Rarely ◯ Never

4. In your opinion or experience, which of the following characteristics often apply to patients affected by somatoform physical complaints/disorders? (More than one can be stated)

◯ Patients ask many questions

◯ Hypersensitivity, patients are easily irritated, quickly offended

◯ Overworked, occupational stress

◯ Patients are easily influenced by other sources of information, e.g. by internet research

◯ Patients are aggressive, ready for conflict

◯ Patients are anxious, nervous, easily get worked up

◯ Patients are frequently affected by psychological disorders such as depression, panic or obsessive-compulsive disorders

◯ Patients come for consultation with false expectations or assumptions (e.g. information consulted, general practitioner role)

◯ Patients seek medical consultation more often than the average

◯ Patients are more critical towards me as a doctor

◯ Patients have little trust, are sceptical

◯ Patients have an exaggerated use of medication

◯ Patients often request further instrument-based diagnostics

◯ It is difficult to distract patients from their opinions, worries or concerns, even if they are proven to be unjustified

◯ These patients lack realisation of their actual condition

◯ Patients tend to imagine physical complaints

◯ Patients tend to practice self-medication

◯ Patients quickly break off the contact with the doctor if their expectations are disappointed and seek new doctors ('doctor hopping')

◯ Patients suffer from a chronic disease

5. When you consider your patients who are affected by somatoform physical complaints/disorders: In your estimation or experience, what are the common causes of such complaints? (More than one can be stated)

◯ Unfavourable life circumstances in childhood and early life phases (e.g. violence, exclusion, harassment)

◯ Chronic or severe physical illnesses

◯ Extraordinary burdens (e.g. unemployment, separation, accidents, operations, loss of a close person)

◯ Social conflicts, lack of social support

◯ Stress factors and conflicts at work

◯ Worrying about physical complaints, hypochondria

◯ Changes in life circumstances (e.g. career changes, retirement, moving home, giving birth)

◯ Biological and genetic factors

◯ Other, namely:

____

6. On what basis or according to which terminological diagnostic reference do you usually diagnose non-specific and somatoform physical complaints?

____

____

7. ICD-10 provides different diagnostic references for non-specific and somatoform physical complaints. How often do you work with the ICD code for this condition for coding/classification purposes?

◯ Frequently ◯ Occasionally ◯ Rarely ◯ Never

- If 'Yes': Please go directly to question 9:

8. Why do you tend not to use or do not regularly use the ICD code for these symptoms?

____

____

9. Do you use certain tools such as (psychosomatic) checklists or questionnaires for diagnosis or for differential diagnostic considerations of these symptoms?

◯ (Psychosomatic) checklist(s) ◯ Questionnaires

◯ Other, namely: ____

10. How demanding or challenging do you find the care and treatment of patients with non-specific somatoform physical complaints in your daily practice?

◯ Very strenuous ◯ Rather strenuous ◯ Less strenuous ◯ Not strenuous at all
◯ Don't know

11. Treating doctors may be faced with challenges in the care and treatment of patients with non-specific somatoform physical complaints/disorders. How great do you experience the following potential challenges when you think about your previous experiences with these patients?

Providing sufficient time for these patients
◯ Very challenging ◯ Rather challenging ◯ Less challenging ◯ Not at all challenging

Counteracting or eliminating concerns or fears of a possible illness
◯ Very challenging ◯ Rather challenging ◯ Less challenging ◯ Not at all challenging

Responding to all questions and wishes of these patients (e.g. with regard to instrument-based diagnostics).
◯ Very challenging ◯ Rather challenging ◯ Less challenging ◯ Not at all challenging

Providing patients with a realistic picture of the possibilities and limitations of medical diagnostics and/or therapy.
◯ Very challenging ◯ Rather challenging ◯ Less challenging ◯ Not at all challenging

Avoiding or eliminating misunderstandings and disappointments on the part of the patients
◯ Very challenging ◯ Rather challenging ◯ Less challenging ◯ Not at all challenging

Ensuring compliance
◯ Very challenging ◯ Rather challenging ◯ Less challenging ◯ Not at all challenging

Encouraging patients to make use of psychosocial support services (e.g. psychotherapy, resilience training).
◯ Very challenging ◯ Rather challenging ◯ Less challenging ◯ Not at all challenging

Other, namely: ______________________
◯ Very challenging ◯ Rather challenging ◯ Less challenging ◯ Not at all challenging

12. Here are various imaginable approaches that the general practitioner can apply to stabilise patients with non-specific somatoform physical complaints/disorders or to have a positive influence on them, so that the doctor-patient relationship also benefits from this. Which of these have you already applied and experienced a good result with?

◯ Tangential dialogue: Doctor-patient dialogue follows the report of the patient's complaints; the patient is given space to explain; confrontational dialogue techniques are avoided; radiation of a calm, objective and attitude giving patients the feeling that they are being taken seriously.

◯ Assurance of the credibility of the complaints

◯ Getting to know the patient to enable the assessment of his/her personality

◯ Avoidance of inciting exaggerated expectations regarding diagnostics and therapy; dampening of over-optimistic patient expectations

◯ Careful marking of references to psychosocial problems as relevant and addressing them in a casual manner (“stress”, “strain” etc.)

◯ Refraining from negative wording of diagnostic findings (“You aren’t ill”)

◯ Long-term building of motivation for holistic treatment if possible

◯ Provision of more consultation time (e.g. detailed explanation, emotional support)

◯ Scheduling of regular appointments (time-contingent instead of complaint-driven)

◯ Formulation of realistic, specific and verifiable therapy goals (improvement of quality of life instead of targeting complete cure)

◯ Targeting in intermediate steps; not too many goals in too short a time

◯ Use of selected information material to successively communicate to the patient that there are symptoms without a clear physical origin

◯ Diary of complaints or anxieties: ◯ When do complaints occur and in what way?

◯ Prescription of supporting measures, e.g. procedures for relaxation and stress management, mindfulness training

◯ Dosed physical activity to change body awareness (reduction of fear and loss of control)

◯ Physiotherapy services

◯ Referral to low-threshold psychosocial or psychotherapeutic services (e.g. special services offered by health insurance companies)

◯ Referral or arrangement of psychosocial support services (e.g. consultation centres, self-help groups)

◯ Referral of the patient to a psychotherapist

◯ Medication therapy preferably only for pronounced comorbidities

13. Are you familiar with the S3 guideline "Management of patients with non-specific, functional and somatoform physical complaints"?

◯ Yes, I am familiar with it ◯ No, I am not familiar with it => **go directly to question 15**

14. What would you say: How familiar are you with this guideline?

◯ Very familiar ◯ Rather familiar ◯ Less familiar ◯ Not at all familiar

15. How useful or helpful do you find this guideline in the diagnosis and treatment of patients with non-specific somatoform physical complaints/disorders?

◯ Very helpful ◯ Rather helpful ◯ Less helpful ◯ Not at all helpful

16. Why do you find the guideline (rather) helpful or less helpful or not helpful at all?

____

____

17. How confident do you feel when it comes to managing non-specific somatoform physical complaints/disorders, i.e. accompanying patients with therapeutic and, if necessary, medicinal measures?

◯ Very confident ◯ Rather confident ◯ Less confident ◯ Not confident at all

18. From your own experience, how would you assess the cooperation between general practitioners and regional physicians (psychiatry/psychosomatics, neurology) when it comes to the treatment of somatoform physical complaints/disorders?

◯ Very good ◯ Rather good ◯ Rather poor ◯ Very poor ◯ Hard to say

19. How do you assess from your own experience the cooperation between GPs and psychotherapists when it comes to the treatment of somatoform physical complaints/disorders?

◯ Very good ◯ Rather good ◯ Rather poor ◯ Very poor ◯ Hard to say

20. Which of the following statements do you agree with in your experience?

"I can rely on the effective support of psychiatrists and psychotherapists in the treatment of somatoform physical complaints".

◯ Strongly agree ◯ Somewhat agree ◯ Somewhat disagree ◯ Not at all agree
◯ Hard to say

"The colleagues in psychiatry and psychotherapy usually inform me sufficiently about the examinations carried out, results and/or therapeutic measures."

◯ Strongly agree ◯ Somewhat agree ◯ Somewhat disagree ◯ Not at all agree
◯ Hard to say

"Due to the long waiting times for psychiatrists and psychotherapists, I am generally rather reluctant to refer patients to them."

◯ Strongly agree ◯ Somewhat agree ◯ Somewhat disagree ◯ Not at all agree
◯ Hard to say

"When it comes to the management of patients with somatoform complaints, I often feel left alone in my work as a general practitioner."

◯ Strongly agree ◯ Somewhat agree ◯ Somewhat disagree ◯ Not at all agree
◯ Hard to say

21. In your opinion, what would need to change in order for somatoform disorders to be better managed and treated in GP practices? Please limit yourself to three points at the most.

____

____

____

Now we need some statistical information from you. As with the rest of the questionnaire, this information will of course be kept strictly confidential and anonymised.

You are…

◯ Male ◯ Female ◯ Diverse

Your **age**: _____

You are a **specialist for**...

◯ General medicine ◯ Internal medicine (as a general practitioner) ◯ Other

Where is your practice? In a municipality/town/city with ...

◯ more than 100,000 ◯ 20,000-100,000 ◯ 5,000 to 20,000 ◯ less than 5,000 inhabitants

Which model best describes your practice?

◯ Individual practice ◯ Individual practice with employed doctors

◯ Joint practice ◯ Group practice

◯ Other

How many patients does your practice treat per quarter?

◯ 500-750 ◯ 751-1000 ◯ 1001-1500 ◯ 1501-2000 ◯ More than 2000

Thank you for your participation!

Is there anything else you would like to tell us?
Here you will find space for suggestions, comments and criticism.

____

____

____
